# The Unified Multiple System Atrophy Rating Scale: Status, Critique, and Recommendations

**DOI:** 10.1002/mds.29215

**Published:** 2022-09-08

**Authors:** Florian Krismer, Jose‐Alberto Palma, Giovanna Calandra‐Buonaura, Iva Stankovic, Luca Vignatelli, Anna‐Karin Berger, Cristian Falup‐Pecurariu, Alexandra Foubert‐Samier, Günter Höglinger, Horacio Kaufmann, Larry Kellerman, Han‐Joon Kim, Thomas Klockgether, Johannes Levin, Pablo Martinez‐Martin, Tiago A. Mestre, Maria Teresa Pellecchia, Susan Perlman, Irfan Qureshi, Olivier Rascol, Anette Schrag, Klaus Seppi, Huifang Shang, Glenn T. Stebbins, Gregor K. Wenning, Wolfgang Singer, Wassilios G. Meissner

**Affiliations:** ^1^ Department of Neurology Medical University Innsbruck Innsbruck Austria; ^2^ Department of Neurology New York University Grossman School of Medicine New York New York USA; ^3^ Dipartimento di Scienze Biomediche e Neuromotorie Università di Bologna Bologna Italy; ^4^ IRCCS, Istituto delle Scienze Neurologiche di Bologna Bologna Italy; ^5^ Neurology Clinic, Clinical Center of Serbia, Faculty of Medicine University of Belgrade Beograd Serbia; ^6^ Clinical Science, Assessment and Innovation, Department of Clinical Development Lundbeck Valby Denmark; ^7^ Department of Neurology, County Clinic Hospital, Faculty of Medicine Transylvania University Brasov Romania; ^8^ CHU Bordeaux, Service de Neurologie des Maladies Neurodégénératives, IMNc Bordeaux France; ^9^ German Center for Neurodegenerative Diseases (DZNE) Munich Germany; ^10^ Department of Neurology Hannover Medical School Hannover Germany; ^11^ Patient Advocacy Group Multiple System Atrophy Coalition McLean Virginia USA; ^12^ Department of Neurology and Movement Disorder Center Seoul National University Hospital Seoul South Korea; ^13^ Department of Neurology University Hospital Bonn Bonn Germany; ^14^ German Center for Neurodegenerative Diseases (DZNE) Bonn Germany; ^15^ Department of Neurology Ludwig‐Maximilians‐Universität München Munich Germany; ^16^ Munich Cluster for Systems Neurology (SyNergy) Munich Germany; ^17^ SME MODAG GmbH Wendelsheim Germany; ^18^ Center for Networked Biomedical Research in Neurodegenerative Diseases (CIBERNED) Carlos III Institute of Health Madrid Spain; ^19^ Parkinson's Disease and Movement Disorders Center, Division of Neurology, Department of Medicine The Ottawa Hospital Research Institute, University of Ottawa Brain and Mind Institute Ottawa Ontario Canada; ^20^ Department of Medicine, Surgery and Dentistry, Neuroscience Section University of Salerno Salerno Italy; ^21^ Department of Neurology David Geffen School of Medicine at UCLA Los Angeles California USA; ^22^ Pharmaceutical Company Biohaven Pharmaceuticals New Haven Connecticut USA; ^23^ French Reference Center for MSA, CIC 1436, NS‐Park/FCRIN network and NeuroToul COEN Center University Hospital of Toulouse, University of Toulouse 3 and INSERM Toulouse France; ^24^ Department of Clinical Neurosciences, UCL Queen Square Institute of Neurology University College London London United Kingdom; ^25^ Department of Neurology, Laboratory of Neurodegenerative Disorders, Rare Diseases Center West China Hospital, Sichuan University Chengdu China; ^26^ Department of Neurological Sciences Rush University Medical Center Chicago Illinois USA; ^27^ Department of Neurology Mayo Clinic Rochester Minnesota USA; ^28^ University of Bordeaux, CNRS, IMN, UMR 5293 Bordeaux France; ^29^ Department of Medicine University of Otago Christchurch New Zealand; ^30^ New Zealand Brain Research Institute Christchurch New Zealand

The Unified Multiple System Atrophy Rating Scale (UMSARS) was developed almost two decades ago as a clinical rating scale to capture multiple aspects of the disease.^1^, ^2^ It is composed of four subscales: UMSARS‐I (12 items) rates patient‐reported functional disability, UMSARS‐II (14 items) assesses motor impairment based on a clinical examination, UMSARS‐III records blood pressure and heart rate in the supine and standing positions, and UMSARS‐IV (1 item) rates chore‐based disability. Higher scores on the UMSARS indicate greater disability. Since its development and validation, the UMSARS has been widely used, in particular as an endpoint of clinical trials and academic research.^3^, ^4^, ^5^, ^6^, ^7^, ^8^, ^9^, ^10^, ^11^, ^12^

With its increasing use, potential areas of improvement in the UMSARS have become apparent. We here address the limitations of the UMSARS and suggest a framework to develop an improved multiple system atrophy (MSA) clinical outcome assessment. To this end, a task force, involving clinicians, researchers, patient support groups, and industry representatives, has recently been endorsed by the International Parkinson's Disease and Movement Disorder Society (MDS).

## Development and Validation of the UMSARS

The UMSARS was developed in the early 2000s by the European MSA Study Group recognizing the need for developing a disease‐specific rating instrument. These efforts were driven by previous studies demonstrating that the clinical rating scales available at the time did not adequately capture MSA‐specific symptoms.^13^, ^14^ The UMSARS was clinimetrically validated in 40 patients with MSA, and the validation included interrater and intrarater reliability assessment of each item, evaluation of its internal consistency, and construct validity confirmation.^1^, ^2^ Although overall the UMSARS had a good clinimetric profile, it was evident that some items had limitations. All but one UMSARS‐I item (item 9, orthostatic symptoms) showed substantial to excellent interrater agreement. A subsequent analysis of the intrarater agreement found that all of the UMSARS‐II items had substantial or excellent intrarater reliability, except for oculomotor dysfunction (item 3), which had moderate intrarater agreement.^2^ Internal consistency was overall high; however, UMSARS‐I items 8 (falling) and 9 (orthostatic symptoms), as well as UMSARS II item 3 (oculomotor dysfunction), correlated poorly with the subscale's sum score. Analysis of criterion‐related validity of the UMSARS demonstrated a strong correlation between UMSARS‐I, UMARS‐II, and a three‐point overall severity scale (categorizing disease severity to mild, moderate, or severe). Content validity was confirmed by a strong correlation between the UMSARS and the Unified Parkinson's Disease Rating Scale, as well as between the UMSARS‐II and the International Cooperative Ataxia Rating Scale.^1^

Notably, UMSARS was validated in a cohort of moderately to severely disabled patients; hence it remains unclear if the results of the validation also apply to patients with mild severity.^1^ Furthermore, the clinimetric validation lacked a formal assessment of floor and ceiling effects, and the latter may partially explain observations from natural history studies showing a faster progression of UMSARS in patients with early disease.^3^, ^5^ Despite the good to excellent intrarater and interrater reliability,^1^, ^2^ imperfect scoring instructions and anchoring descriptions may contribute to some scoring inconsistencies. Additional limitations include the redundancy of items assessing similar functions through patient reports and motor examination, as well as incomplete representation of common and specific features of MSA (eg, mood disorders, stridor). Some features captured in the UMSARS are amenable to symptomatic treatment, and invasive/burdensome treatments are sometimes disregarded as scoring options, which may possibly introduce a scoring bias. Finally, the UMSARS was never formally translated and validated into different languages, with the exception of Japanese,^15^ and cultural differences were not studied, which limits its global applicability. In summary, despite its validity, the clinimetric properties of certain UMSARS items can be improved (Table 1).

**TABLE 1:** Identified limitations of the UMSARS related to different taxonomic properties

Property^a^	Limitation
Content validity (including face validity)	MSA patients' and caregivers' input not considered MSA disease‐specific features not considered (eg, depression, stridor) Instrumental assessment of activities of daily living (other than chores) not considered Lack of association with disease severity in some items (eg, UMSARS‐I item 11 on sexual function) Some items are amenable to symptomatic treatment (eg, UMSARS‐I item 2 on swallowing; UMSARS‐1 item 12 on constipation; UMSARS‐I item 9 on orthostatic hypotension)^b^
Reliability	Moderate intrarater and interrater agreement in some items (eg, UMSARS‐II item 3 on oculomotor dysfunction)
Construct/Structural validity	Redundancy of some items (eg, UMSARS‐I item 7 and UMSARS‐II item 14 on gait are both the same)
Cross‐cultural validity	Cultural bias in some items (eg, UMSARS‐I item 4 on cutting food/handling utensils assumes that food is regularly cut for eating)
Criterion validity	Limitations to detect changes in advanced stages—possible ceiling effect
Responsiveness	Limitations to detect disease progression accurately in early stages—variable standard deviations in annual increase exceeding expected effect size of candidate disease‐modifying drugs
Interpretability	Although interrater reliability is good, anchoring descriptions in some items could be improved (eg, UMSARS‐I item 6 on difficulty with showering, unclear if this includes getting into the shower; UMSARS‐I item 2 on swallowing and UMSARS‐I item 8 on falling include “less than once a week” and “more than once a week” options, but it is unclear which would apply in a patient choking/falling once a week)

^a^ Taxonomy and definition of measurement properties according to Mokkink et al.^25^

^b^ The impact of symptomatic therapies in some scale items is not limited to the UMSARS and affects other clinical outcome assessments used in the movement disorders field. UMSARS, Unified Multiple System Atrophy Rating Scale; MSA, multiple system atrophy.

## Do UMSARS Items Reflect Symptom Severity and Progression?

Natural history studies and randomized therapeutic trials have provided information about the progression of UMSARS and specific disease‐related milestones.^3^, ^4^, ^5^, ^6^, ^7^, ^8^, ^9^, ^10^, ^12^ Table 2 summarizes selected studies. In addition, three recent analyses have assessed the annual change of individual UMSARS items.^16^, ^17^, ^18^ The first concluded that the UMSARS‐I items 9 (orthostatic symptoms) and 12 (bowel function), as well as UMSARS‐II items 4 and 5 (tremor at rest and action tremor), show little ability to detect change. Conversely, items with good sensitivity to change included those assessing dressing and hygiene and posture and gait.^16^ The second study performed an item‐response theory analysis in 557 patients with MSA with a mean follow‐up of 2.3 years.^17^ The majority of items progressed with disease duration and across the different UMSARS‐IV disability stages, except for UMSARS‐I items 9 (orthostatic symptoms), 10 (urinary function), and 11 (sexual function) and UMSARS‐II items 3 (oculomotor dysfunction) and 4 (tremor at rest). Approximately 70% of the scale information was carried by only 11 (of 26) items. The third one analyzed the sensitivity to change and surrogate patient‐centricity measures of the individual UMSARS items in two independent datasets (clinical trial and natural history study).^19^ Like the other two studies, items related to key motor functions were most sensitive to change, while items assessing autonomic symptoms were less sensitive to change. More UMSARS‐I (compared with UMSARS‐II) items were identified to impact the patients' quality of life.^19^

**TABLE 2:** Prospective longitudinal natural history studies and placebo arms of randomized clinical trials reporting baseline and annual change in the UMSARS‐I and UMSARS‐II

Study	N	Mean symptom duration at baseline, years (SD or range)	UMSARS‐I baseline (SD or range)	UMSARS‐I at 1 year (SD or range)	UMSARS‐I difference (SD)	UMSARS‐II baseline (SD or range)	UMSARS‐II at 1 year (SD or range)	UMSARS‐II difference (SD)
**Prospective natural history studies reporting UMSARS‐I and UMSARS‐II**								
Wenning et al.^3^ (European MSA Natural History Study)	87	5.5 (3.8)	25.2 (8.8)	30.9 (9.5)	6.5 (6)	25.9 (9)	33.7 (10)	8.2 (7)
Low et al.^5^ (US MSA Natural History Study)	96	NR	25 (8.1)	26.7 (8.2)	3.9 (5.8)	26.1 (9.3)	28.7 (7.3)	4.6 (5)
Foubert‐Samier et al.^10^ (French MSA Natural History Study)	261	4.5 (1–16)	19.6 (7.3)	23.3 (8.2)	4.9 (5.5)	21.2 (8.0)	25.5 (9.1)	5.4 (6.0)
**Placebo arms of randomized clinical trials**								
Low et al.^4^ (rifampicin trial)	39	NR	12.1 (3.4)	NR	5.6 (5.0)	15.2 (4.8)	NR	5.4 (6.6)
Poewe et al.^6^ (rasagiline trial)	90	Placebo group: 3.7 (2.4) Treatment group: 4.2 (2.4)	16.8 (5.5)	NR	4.4 (0.6)	19.6 (4.9)	NR	3.5 (0.6)
Levin et al.^9^ (PROMESA trial)	45	Placebo group: 3.9 (2.5–4.6) Treatment group: 3.7 (2.9–4.7)	NR	NR	NR	22 (16–27)	NR	6.6 (1)
Meissner et al.^11^ (AFF009 trial)	6	2.8^a^	15.5 (10–27)	23.5 (18–40)	NR	14.5 (12–29)	24.5 (20–43)	NR
Palma et al.^12^ (sirolimus trial)	9	NR^b^	20 (15–24)	NR	5.3 (3.8)	22 (18–26)	NR	6.5 (4.5)

^a^ Time since symptom onset per group: placebo group, 2.9; treatment group PD01A, 2.9; treatment group PD03A, 2.5.

^b^ Time since diagnosis: placebo group, 1.6 (1.1–2.1); treatment group, 0.8 (0.5–1.5).

UMSARS, Unified Multiple System Atrophy Rating Scale; SD, standard deviation; MSA, Multiple System Atrophy; NR, Not reported.

It is highly relevant that UMSARS items evaluating autonomic symptoms have poor ability to detect change, given that autonomic features are strongly correlated with quality of life and life satisfaction in patients with MSA.^18^, ^20^, ^21^, ^22^

## How Relevant Are UMSARS Items to Patients?

No patients or caregivers were involved in the development of the UMSARS; hence it remains unclear how relevant the different UMSARS items are to patients. To understand how the different UMSARS items reflect the patients' perspective and to identify missing symptoms, qualitative studies in which MSA patients provide feedback have been initiated in public–private partnerships. In addition, a number of task force members are involved in an ongoing effort collecting expert opinions on how the different UMSARS items impact quality of life in patients with MSA. These studies will evaluate the relevance of current UMSARS items and help to identify potentially missing items.

## Concerns from Health Authorities When Using UMSARS as an Endpoint in Clinical Trials

Several pharmaceutical companies and academic centers conducting or planning randomized clinical trials in MSA have highlighted the increasing emphasis that the US Food and Drug Administration (FDA) places on patient‐reported outcomes for clinical trials and detailed scoring instructions. Indeed, in the past, the FDA did not accept the current UMSARS as the primary outcome for registrational trials (in particular, the motor examination, UMSARS‐II) and preferred modified versions of the UMSARS. Accordingly, a recently completed industry‐sponsored randomized clinical trial (NCT03952806) used a modified UMSARS score consisting of a subset of the original UMSARS‐I and UMSARS‐II items as the primary endpoint. This score is currently being validated via psychometric analysis and patient interviews (including concept elicitation and cognitive debrief). The regulatory requirement of patient‐centered tools as endpoints for randomized clinical trials illustrates the critical need to include patients and caregivers in the development and validation process of any new clinical outcome assessment for MSA.

## The Coronavirus Disease 2019 Pandemic and the Importance of Virtual Assessments

The current UMSARS, specifically the UMSARS‐II and UMSARS‐III, require in‐person evaluations. The lessons learned from the coronavirus disease 2019 pandemic and the need to increase patient recruitment and retention in clinical trials underline the importance of remote/virtual outcome assessments.^23^ To this end, detailed instructions on how to perform tasks and detailed scoring descriptions are required. Furthermore, the agreement between the in‐person and the virtual assessments must be evaluated.

## The Task Force Roadmap for a Revised MDS‐UMSARS

Recognizing the shortcomings of the current UMSARS, an MDS task force was established to revise the UMSARS and transform it into the MDS‐UMSARS. The MDS‐UMSARS will comprise the following: (1) a comprehensive scale covering the entire spectrum of MSA‐specific symptoms, (2) a patient‐centered scale that satisfies health authorities requirements to be used in therapeutic trials, and (3) a set of virtually assessable items.

To accomplish this, four preparatory steps will be initiated to gather additional information. First, the results of two qualitative, industry‐led studies with patients and caregivers will be considered. These studies include two parts, one to assess the relevance of current UMSARS items and another part to elicit concepts and identify features that are currently not captured by the UMSARS. Second, a structured expert survey will collect experts' perspectives regarding the application and utility of the current UMSARS. Third, data on UMSARS scores collected in natural history studies and clinical trials will be scrutinized to elucidate the clinimetric properties, sensitivity to change, and patient centricity of each item. Fourth, a systematic review will be performed to estimate the frequency and severity of MSA‐specific symptoms.

These early initiatives will inform the drafting of the first iteration of the revised MDS‐UMSARS. The structure of the MDS‐UMSARS might be aligned to the current UMSARS and other validated scales developed for related disorders (eg, the MDS Unified Parkinson's Disease Rating Scale). We anticipate that the final structure of the MDS‐UMSARS will consist of several parts, including a patient/caregiver‐administered questionnaire of motor and nonmotor aspects of daily living, as well as a motor and autonomic examination. The conceptual construct will focus on the impact that the symptoms have on the patient. Each item will be anchored to responses linked to commonly accepted clinical terms, and each item will contain detailed instructions for scoring during in‐person and virtual visits. The first draft will be developed by a core group of task force members, and this draft will be refined through an iterative Delphi process that will eventually define the final structure and overall content of the revised MDS‐UMSARS. The Delphi panel will consist of clinicians, researchers, patient representatives, and a spokesperson from industry.

Field testing of the preliminary new MDS‐UMSARS will then be performed. The preliminary MDS‐UMSARS scale will be distributed to selected MSA research centers in English‐speaking countries. Patients, caregivers, and their treating physicians will be invited to complete the questionnaire. The main purpose of this step will be cognitive debriefing and readability testing. Standard item reduction methods will be applied to develop a clinically meaningful, final iteration of the MDS‐UMSARS. The final version of the revised MDS‐UMSARS will be validated in a multicenter study, including confirmation of construct validity through correlation with relevant other scales/questionnaires, such as the MSA quality of life questionnaire.^24^

Another important goal of this task force is to develop a patient‐centered MDS‐UMSARS to be used as an endpoint for disease modification clinical trials in patients at early disease stages. This scale will be based on MDS‐UMSARS items. Determining the relevance of the selected items for the early disease stage population, addressing ambiguity in scoring, and minimizing redundancy will be key. Before using it as an endpoint, this clinical trial MDS‐UMSARS must have undergone longitudinal validation of its responsiveness to change over the duration of a typical clinical trial (ie, 1 year) in patients at early disease stages.

The earlier‐described guidance by the FDA has led to independent initiatives by several industry sponsors in creating modified versions of UMSARS with the risk of differing scales for the upcoming treatment trials. Because the development of the new abbreviated UMSARS will take several years, the UMSARS task force intends to develop, in collaboration with these industry sponsors, a modified UMSARS for temporary use. This modified version will be based on items of the current UMSARS and rely on available data from previous treatment trials and natural history cohorts. The scale will focus on the sensitivity to change of individual UMSARS items, as well as the association of individual items with quality‐of‐life measurements, and will likely consist of a subset of current UMSARS items that carried most of the scale information on progression in previous studies irrespective of disease duration. This work was initiated in 2018 outside this task force and is expected to be completed by the end of 2022. Outside the MDS‐UMSARS revision core program, the task force will initiate and facilitate additional steps, including non‐English translations, the characterization of its minimal clinically important change, and the development of educational and training materials, including a video tutorial to standardize its administration and scoring.

## Financial Disclosures

F.K.: Received personal fees from Institut de Recherches Internationales Servier, Clarion Healthcare, Takeda Pharmaceuticals, and the Austrian Society of Neurology; grant support from the MSA Coalition outside of the submitted work.

J.‐A.P.: Research funding from the National Institutes of Health (NIH), MSA Coalition, The Michael J. Fox Foundation. Employee of Novartis.

G.C.‐B.: Speaker's honoraria from AbbVie Italia, Bial Italia, and Zambon Italia.

I.S.: None.

L.V.: None.

A.‐K.B.: Employee of Lundbeck.

C.F.‐P.: Editor fees from Springer and Elsevier; speaker's fees from AbbVie and from the International Parkinson and Movement Disorder Society (IPMDS).

A.F.‐S.: Consulting fees from Aguettant Laboratory.

G.H.: None.

H.K.: Research funding from the NIH, MSA Coalition, and The Michael J. Fox Foundation. Advisory board fees from Lundbeck, Takeda, ONO, and Vaxxinity. Editor in Chief of *Clinical Autonomic Research*.

L.K.: None.

H.‐J.K.: Research funding from Seoul National University Hospital, Institute of Information and Communications Technology Planning and Evaluation, Forest Hills, and HLB Pharmaceutical.

T.K.: None.

J.L.: Employee and shareholder of MODAG GmbH. Inventor of patent “Pharmaceutical Composition and Methods of Use” (EP 22159408.8) filed by MODAG GmbH. Speaker's fees from Bayer Vital, Biogen, and Roche; consulting fees from Axon Neuroscience and Biogen; author fees from Thieme medical publishers and W. Kohlhammer GmbH medical publishers.

P.M.‐M.: Editorial fees from Editorial Viguera for lecturing in courses; consulting fees from Bial; and honoraria from the IPMDS.

T.A.M.: Consultancy/Speaker honorarium: AbbVie, IPMDS, AAN, AbbVie, CHDI Foundation/Management, Sunovion, Valeo Pharma, Roche, nQ. Medical advisory board: AbbVie, Biogen, Sunovion, Medtronic. Research funding: EU Joint Programme ‐ Neurodegenerative Disease Research, uOBMRI, Roche, Ontario Research Fund, CIHR, MJFF, Parkinson Canada, PDF/PSG, LesLois Foundation, PSI Foundation, Parkinson Research Consortium, and Brain Canada.

M.T.P.: None.

S.P.: None.

I.Q.: Employee and shareholder of Biohaven Pharmaceuticals.

O.R.: Scientific consultant for drug companies developing new treatments for MSA, including Affiris, Biogen, Biohaven, Lundbeck, ONO, Servier, Takeda, and TEVA; has received scientific grants from the French ministry of health for academic research programs in MSA.

K.S.: Reports personal fees from Teva, UCB, Lundbeck, AOP Orphan Pharmaceuticals AG, Roche, Grünenthal, Stada, Licher Pharma, Biogen, BIAL, and AbbVie; honoraria from the IPMDS; research grants from FWF Austrian Science Fund, The Michael J. Fox Foundation, and AOP Orphan Pharmaceuticals AG, outside the submitted work.

A.S.: Research funding from GE Healthcare; honoraria for consultancy from Biogen, AbbVie, Roche, Bial, GE Healthcare; license fee payments from the University College London for the MSA‐QoL, PSP‐QoL, and PQolCarers; and royalties from Oxford University Press.

H.S.: Research funding from the Sichuan Science and Technology Program (Grant 2022ZDZX0023).

G.T.S.: Consulting and Advisory Board Membership with honoraria: Adamas Pharmaceuticals, Ceregene, Inc., CHDI Management, Inc., Cleveland Clinic Foundation, Neurocrine Biosciences, Inc., Pfizer, Inc., Tools‐4‐Patients. Grants and Research: NIH, Department of Defense, The Michael J. Fox Foundation for Parkinson's Research, Dystonia Coalition, CHDI, IPMDS, Columbia University. Honoraria: IPMDS, American Academy of Neurology, The Michael J. Fox Foundation for Parkinson's Research, FDA, NIH, and Alzheimer's Association.

G.K.W. reports consultancy and lecture fees from AbbVie, Affiris, AstraZeneca, Biogen, Biohaven, Inhibicase, Lundbeck, Merz, Novartis, Ono, Teva, and Theravance; research grants from the FWF Austrian Science Fund, the Austrian National Bank, the US MSA Coalition, Parkinson Fonds Austria, and IPMDS, outside of the submitted work.

W.S.: None.

W.G.M.: Fees for editorial activities with Elsevier, consultancy for Lundbeck, Biohaven, Roche, Alterity, Servier, Inhibikase, Takeda, and Teva, and teaching honoraria from UCB.

## Author Roles

1. Research project: A. Conception, B. Organization, C. Execution;

2. Statistical Analysis: A. Design, B. Execution, C. Review and Critique;

3. Manuscript Preparation: A. Writing of the first draft, B. Review and Critique.

F.K.: 1B, 1C, 3A.

J.‐A.P.: 1B, 1C, 3A. G.C.‐B.: 1C, 3B.

I.S.: 1C, 3B.

L.V.: 1C, 3B.

A.‐K.B.: 1C, 3B.

C.F.‐P.: 1C, 3B.

A.F.‐S.: 1C, 3B.

G.H.: 1C, 3B.

H.K.: 1C, 3B.

L.K.: 1C, 3B.

H.‐J.K.: 1C, 3B.

T.K.: 1C, 3B.

J.L.: 1C, 3B.

P.M.‐M.: 1C, 3B.

T.A.M.: 1C, 3B.

M.T.P.: 1C, 3B.

S.P.: 1C, 3B.

I.Q.: 1C, 3B.

O.R.: 1C, 3B.

K.S.: 1C, 3B.

A.S.: 1C, 3B.

H.S.: 1C, 3B.

G.T.S.: 1C, 3B.

G.K.W.: 1C, 3B.

W.S.: 1A, 1B, 1C, 3B.

W.G.M.: 1A, 1B, 1C, 3B.

## Data Availability

Data sharing not applicable ‐ no new data generated, or the article describes entirely theoretical research
